# Association of Dietary Glycemic Index, Glycemic Load, Insulin Index, and Insulin Load with Bacterial Vaginosis in Iranian Women: A Case-Control Study

**DOI:** 10.1155/2022/1225544

**Published:** 2022-03-24

**Authors:** Morvarid Noormohammadi, Ghazaleh Eslamian, Seyyedeh Neda Kazemi, Bahram Rashidkhani, Shirin Malek

**Affiliations:** ^1^Department of Nutrition, School of Public Health, Iran University of Medical Sciences, Tehran, Iran; ^2^Department of Cellular and Molecular Nutrition, Faculty of Nutrition and Food Technology, National Nutrition and Food Technology Research Institute, Shahid Beheshti University of Medical Sciences, Tehran, Iran; ^3^Department of Obstetrics and Gynecology, School of Medicine, Preventative Gynecology Research Center, Imam Hossein Hospital, Shahid Beheshti University of Medical Sciences, Tehran, Iran; ^4^Department of Community Nutrition, Faculty of Nutrition and Food Technology, National Nutrition and Food Technology Research Institute, Shahid Beheshti University of Medical Sciences, Tehran, Iran; ^5^Department of Nutrition and Food Science, California State University, Chico, CA 95929, USA

## Abstract

**Background:**

Inconsistent findings have been reported for associations between dietary indices and bacterial vaginosis (BV). The aim of this study was to examine the association of dietary glycemic index (DGI), glycemic load (DGL), insulin index (DII), and insulin load (DIL) with BV among Iranian women.

**Methods:**

The current case-control study consisted of 144 new cases of BV and 151 controls. The diagnosis of BV was made based on the Amsel criterion in hospital clinics in Tehran, Iran, from November 2020 until June 2021. DGI, DGL, DII, and DIL were calculated from a validated semiquantitative food frequency questionnaire. The association between dietary carbohydrate indices and odds of BV were assessed adjusting for potential confounders through an estimation of two multivariate regression models.

**Results:**

The multivariate adjusted odds ratio (OR) comparing the highest tertile of dietary DGI and DGL with the lower tertile was 2.99 (95% confidence interval (CI): 1.47–6.81; *P*_trend_ = 0.003) and 4.01 (95% CI: 1.22–5.91; *P*_trend_ = 0.029), respectively. In a fully adjusted model, the top tertile of dietary fiber compared to the bottom was associated with 88% (95% CI: 0.14-0.33) lower odds of BV (*P*_trend_ < 0.001). DII and DIL were not significantly associated with odds of BV in both crude and adjusted regression models.

**Conclusion:**

The findings support the hypothesis of moderate, direct associations between DGI or DGL and BV. Also, a diet high in fiber decreases odds of BV.

## 1. Introduction

Bacterial vaginosis (BV) is the most prevalent gynecological disorder [1], estimated to affect 29% of women of reproductive age [2]. The overall prevalence of BV among nonpregnant Iranian women was 28% [3]. BV is defined by a switch in the vaginal flora from the dominant *Lactobacillus* to a polymicrobial flora including *Gardnerella*, *Atopobium*, and *Prevotella* [4]. Symptoms like vaginal discharge, itching, and burning commonly occur in BV without redness and swelling [5]. This vaginal condition leads to several problems, including preterm birth, endometritis, and increased risk of sexual transmitted diseases [1]. BV risk factors include smoking, douching, multiple sexual partners, and using intrauterine devices [2]. BV is a difficult-to-treat disease [6]. Half of BV patients may be affected again after medical treatment in the following 12 months [7]. Despite the high prevalence of BV and adverse reproductive and obstetric health outcomes, there is little information about its diet-risk factors.

Dietary intake may affect vaginal flora imbalances. Previous studies have illustrated a link between BV and diet and nutritional status [8–12]. Only one study has examined the association of dietary glycemic index (DGI) and dietary glycemic load (DGL) with BV to the best of our knowledge. Thoma et al. reported a positive association between DGL and BV progression and BV persistence [8]. There is emerging evidence that dietary carbohydrate indices are a reliable tool based on the differing physiological response to carbohydrate-containing foods [13]. Carbohydrate-containing foods increase postprandial serum glucose and insulin secretion at different rates based on the type of the carbohydrates, the amount and type of fiber contained, and the processing method. These differences are indicated by the DGI [13]. DGI is a ranking system to estimate the insulinogenic effects of foods. High-GI diets have been shown as the etiology of many chronic diseases [14, 15]. DGL estimates the serum glucose response using the DGI, and the quantity of carbohydrate-containing foods consumed [16]. DGL equals the DGI multiplied by the amount of available carbohydrates divided by 100 [15]. However, the DGI concept does not apply to low-carbohydrate and carbohydrate-free foods.

Moreover, other factors in combination with carbohydrates synergistically impact insulin secretion, including specific amino acids, fructose, and fatty acids. Accordingly, the dietary insulin index (DII) is a concept to investigate the insulin response of healthy people after consumption of a specific food in comparison with an isoenergetic portion of a reference food (analogous to the DGI concept glucose or white bread) [17]. On the basis of the DII, the dietary insulin load (DIL) can be estimated by multiplying the DII of each food item by its consumption frequency and energy content [18].

Therefore, there is a shortage of data on the relationship between dietary carbohydrate indices and BV. Thus, this finding encouraged us to design a case-control study to evaluate the association of DGI, DGL, DII, and DIL with BV among Iranian women.

## 2. Materials and Methods

### 2.1. Ethical Considerations

All procedures were conducted according to the latest version of the Helsinki Declaration. The study was approved by the ethics committee of the National Nutrition and Food Technology Research Institute, Shahid Beheshti University of Medical Sciences, Tehran, Iran (IR.SBMU.NNFTRI.REC.1399.054). All BV cases and controls signed a consent form.

### 2.2. Study Population

In this hospital-based case-control study, 148 women with incident BV and 153 controls were selected by convenience sampling method among the Tehran-resident patients referred to the gynecology clinic at Imam Hossein Hospital in Tehran, Iran, from November 2020 until June 2021. A gynecologist examined all participants to evaluate for bacterial vaginosis. According to the Amsel criteria, newly diagnosed BV patients (identified within three months of diagnosis) had to have at least three out of four symptoms, including homogeneous and dilute vaginal discharge, vaginal pH greater than 4.5, the presence of 20% of clue cells under saline microscopy, and fish odor after adding 10% potassium hydroxide to the discharge slide [19]. The inclusion criteria for cases were women diagnosed with BV, age group of 18-45 years, no pregnancy, no menopause, no use of antibiotics, probiotics, hormonal contraceptives, vaginal douches and immunosuppressive drugs, no systemic immunity diseases, chronic infection, chronic diet-related diseases (diabetes, cardiovascular disease, etc.), or any disease in the uterine cavity such as polyp and fibroids as well as lack of hysterectomy. The inclusion criteria for control were not having ongoing or previous BV or BV treatment. The rest of the inclusion criteria for the control group were similar to the case group. The exclusion criteria for both BV cases and controls during the study were as follows: reported energy intakes were outside of the range of ±3 standard deviation (SD) from the average energy intakes of the study population and the participant's inability to respond to the questions.

### 2.3. Sociodemographic Assessment

Age, smoking, BV family history, education level, occupational status, number of sex partners, medication history, supplementation, and monthly family income were recorded. Due to Iranian religious and cultural beliefs, questions regarding alcohol and opium were omitted.

### 2.4. Nondietary Exposure Assessment

Weight, height, and waist circumference (WC) were measured. Weight with light clothing and accuracy of 100 grams and height using a tape measure in a standing and straight position using a ruler placed on the person's head, without shoes, and while the shoulders are in a normal position, with an accuracy of one millimeter. BMI was calculated by dividing weight (kg) by height squared (square meters).WC was measured at the umbilical site, at the abdominal level, over light clothing, in a standing position, without any pressure to the body surface, using an unstretched centimeter tape to the nearest 0.1 centimeters. One trained examiner carried all anthropometric measurements for cases and controls to avoid random observer error. Physical activity was determined using a valid and reliable questionnaire [20].

### 2.5. Dietary Intake Assessment

Trained dietitians collected dietary data using a valid and reliable semiquantitative food frequency questionnaire (FFQ) which estimated dietary intakes during the past year before the diagnosis of BV for cases and one year before the interview in controls. This FFQ is a valid and reliable questionnaire [21] containing 168 food items with a standard serving size for each food item and designed according to the Willet method [22]. During the interview, the average size of each food item in the FFQ was explained to the participants, and then they were asked about the frequency of consumption of each food item in the questionnaire. After completing the FFQ, the mentioned values of each food were converted to grams using the home scale guide. Standard restaurant recipes were used to calculate mixed food items' energy value and nutrient content. The mean daily intakes of energy and macronutrients for each participant were assessed using the USDA food composition table [23] or the Iranian food composition table [24].

### 2.6. Calculation of Carbohydrate Indices

GI values for foods (using glucose as the reference food) were used from the Iranian Food Table of GI and GL [25] and International Tables of GI and GL Values [26]. The missing GI values in the databases were either estimated using both the GI of foods with similar nutritional composition and preparation methods or were calculated using recipes. The food insulin index (FII) represents the incremental insulin concentration in blood 2 hours after consumption of a 1,000 kJ or 239 kcal of a test food. FII was obtained from previous studies [17, 27–29]. For food items that were not listed in the previous studies, the FII value of similar food items was used based on the correlation between their energy, fiber, carbohydrate, protein, and fat .

Dietary carbohydrate indices including DGI, DGL, DII, and DIL were derived from the FFQ as follows:

Dietary glycemic index (DGI) = [(carbohydrate content of each food item) × (number of servings/d) × (glycemic index)]/total daily carbohydrate intake

Dietary glycemic load (DGL) = (carbohydrate content of each food item) × (number of servings/d) × (glycemic index)

Dietary insulin load (DIL) = [*Σ* (food insulin index × kilocalories per gram × gram per day)]

Dietary insulin index (DII) = dietary insulin load/{*Σ* [energy content of food (Kcal/serving) × frequency of consumption (servings of food/day)}

### 2.7. Statistical Analysis

Statistical data analysis was conducted using SPSS version 20.0 (SPSS, Inc.). All hypothesis tests were 2-tailed, with *P* values <0.05 considered statistically significant. Histograms, *Q*–*Q* plots, and the Shapiro–Wilk statistic test were used to determine data normality of continuous variables. General characteristics of cases and controls were expressed as frequency and percentages for qualitative variables and mean (standard deviation [SD]) or median (interquartile range [IQR]) for quantitative variables. Chi-squared test was used to check differences in the distribution of categorical variables (e.g., familial history of BV). Independent *t*-test or Mann–Whitney tests were used to assess differences in the distribution of continuous variables (e.g., age).

Carbohydrate indices were categorized according to the tertile values for each index in the control group. Multiple binary logistic regression was used to determine the odds ratio (OR) with 95% confidence interval (CI) of BV by higher tertile on each carbohydrate index (above tertile vs. below tertile). The first model was adjusted for age, and the second model included the potential confounding variables: age (year), BMI (kg/m^2^), WC (cm), total energy intakes (kcal/d), total fat intake (g/d), physical activity (MET/h/d), and BV family history (yes or no). Analysis of trends was performed by including the categorical variables as continuous predictors in all logistic regression models.

## 3. Results

After calculating the energy intakes of participants, four individuals of the case group and two individuals of the control group whose log scale of total energy intake was either > +3SD or < -3SD from the mean were excluded from the statistical analysis. Participation rates were 97.3% among cases and 98.7% among controls.

General characteristics of the cases (*n* = 144) and controls (*n* = 151) are reported in Table 1. The mean (SD) age of participants was 30.10 (6.03) and 31.44 (7.55) years in cases and controls, respectively, demonstrating the frequency-matching design. Cases had significantly higher BMI (26.29 vs. 25.42) and WC (83.23 vs. 81.42). Incidence of BV within their families was significantly higher in cases than in controls. There was also a significant difference in smoking status between cases and controls. No statistically significant differences were found regarding other demographic and lifestyle exposures between cases and controls.

The median (IQR) of dietary intake and carbohydrate indices for cases and controls are listed in Table 2. Median intakes of fat and DGI were significantly higher among BV cases than controls. On the other hand, the controls consumed significantly more dietary fiber than in cases.

Table 3 shows the association between dietary carbohydrate indices and BV odds. DGI and DGL were significantly associated with BV. The fully adjusted ORs comparing the highest tertile of DGI and DGL with the lowest tertile were 2.99 (95% CI =1.47–6.08; *P* test for trend = 0.003) and 4.01 (95% CI =1.2–5.91; *P* test for trend = 0.029), respectively, with a significant trend. After adjusting for potential confounders, the highest tertile of dietary fiber was associated with a lower odds of BV (OR: 0.22, 95% CI: 0.14–0.33, *P* test for trend < 0.001). DII and DIL were not significantly associated with odds of BV in both crude and adjusted regression models.

## 4. Discussion

To the best of our knowledge, the present study is the first to evaluate the association between dietary indices of carbohydrate quality (DGI, DGL, DII, and DIL) and BV in a developing country. The study demonstrated an association between overall diet and carbohydrate quality with BV, whereas most previous literature assessed individual macro or micronutrients of the overall diet. In this hospital-based case-control study, greater adherence to high-GI/GL diet and a low-fiber diet was significantly associated with increased odds of BV after adjustment for other covariates.

Increasingly, dietary indices rather than single nutrients are being recognized as an important approach to assess eating patterns and behaviors as individuals do not consume nutrients or food in isolation. Diet quality indices are based on scientific facts and determine adherence to dietary guidelines, which are considered important for ensuring an optimal state of health and reflect risk gradients for diet-related noncommunicable diseases (NCDs) [30]. Therefore, dietary indices may be more useful assessment tools because they address a high degree of intercorrelation of nutrients or food groups and provide a useful target for dietary interventions. Thus, dietary indices such as DGI, DGL, DII, and DIL may develop a better understanding of the association between dietary intakes and BV to draw up specific effective prevention strategies.

Emerging evidence indicates that DGI and DGL are reliable dietary assessment tools that consider both amount and source of carbohydrate in the whole diet and may be a better predictor between dietary carbohydrate intake and NCDs but neglect the insulin response [31]. Thus, considering glycemic response along with insulinemic response appears to be more appropriate. Therefore, DII based on the insulin response may be more applicable to test hypotheses linking insulin exposure with diet-related NCDs risk [17].

Consistent with previous studies that show an association between nutritional status and BV, participants in the current study with a healthier diet had a lower likelihood of BV [8–12, 32, 33]. In a study conducted by Thoma et al., DGL was associated with higher BV incidences and increased BV persistence and acquisition [8]. Despite the consistency between the present study results and that of the study by Thoma et al., the results of these studies on the association of the DGI with BV are contradictory. The current study showed that DGI was directly associated with BV at a significant level. In Thoma et al.'s study, DGI was not significantly associated with BV odds [8]. Shivakoti et al. reported that the diets richer in fiber were associated with lower odds of molecular-BV [34]; these data align with the results.

Studies have reported that women with diabetes or gestational diabetes who have poor glycemic control are more likely to be affected by genital tract infections [35, 36]. Several mechanisms have been proposed to explain the effect of source and amount of carbohydrate on BV. The vaginal microecological environment is affected by a number of factors, and it is exposed to dynamic alteration [37]. In an in vitro experimental study, Mirmonsef et al. showed that pH values and colonization by various *Lactobacillus* species could be affected by free glycogen concentration in vaginal fluids [38]. Diets rich in fiber could influence the microbiota towards more *Lactobacillus*-dominant profiles and have a beneficial effect on vaginal and cervical health [34]. The potential for this association is also supported by findings from another *in vitro* study reporting that prebiotic dietary fibers stimulate the growth of monocultures of the major *Lactobacillus* species dominating the vaginal microbial population [39].

On the other hand, diet may affect the microbiota of mucosal surfaces in the reproductive and gastrointestinal tracts. Crescenzo et al. showed that a carbohydrate-rich diet might stimulate alterations in the gut microbiota in rats [40]. Consequently, bacterial colonization of the gut could act as a pool for vaginal microbiota. Antonio et al. reported similarity between *Lactobacillus* species in the vagina and rectum, showing that the rectum could serve as a likely source for vaginal colonization [41]. Continuous exposures to postprandial hyperglycemia may induce oxidative stress through increases in inflammation and reduced plasma antioxidant activity due to free radical production [42]. It is possible that chronic exposure of high-GI/GL diets could influence host response to bacterial colonization and, in particular, the pathogenesis of BV through oxidative damage and impaired immune response.

This study has several strengths. The high participation rate, use of FFQ, and detailed assessment with adjustment for potential confounders were some of the strengths. An advantage of using FFQ over a single 24 h dietary recall is the detailed list of rarely consumed and seasonal food items to collect. In order to control the selection bias, the person performing the diagnostic BV test was not aware of the exposure conditions (food intakes of participants). In addition, to control recall bias, the cases were selected from patients who had been newly diagnosed with this BV in the past three months. A trained dietician completed the questionnaires and was not aware of the outcome of the participants at the time of the interview to control information bias. A person in the hospital laboratory also performed diagnostic tests. Another strength was the several controlled confounding variables. However, this study has several limitations. Selection bias and recall bias in case-control design might result in misleading findings. Since data on exposure and outcome in case-control studies gather simultaneously at one particular time point, causality cannot be conferred. However, since newly diagnosed patients for the case group were selected, there is less concern about this issue in this study. Although the validity and reproducibility of FFQ amongst Iranians have been well supported, the data may suffer from measurement errors. However, participants who under/overreported their energy intakes were excluded. Alcohol consumption was not assessed as a result of its cultural and religious ban and was not included in the analysis. The reason that the study did not show a significant association between DII or DIL and BV may be due to the small sample size, which may have limited power to detect potentially significant associations. The current study has not considered different types of bacteria causing the BV. Finally, since the sample population was women referred to the hospital, they could not represent the target population, women with bacterial vaginosis, which is a challenge in terms of generalizability of results.

## 5. Conclusion

To conclude, the results showed that a low-GI/GL diet and a high-fiber diet might play a role in reducing the odds of BV. Further extensive prospective studies are needed to expand on these findings and evaluate causal links.

## Figures and Tables

**Table 1:** General characteristics of participants with bacterial vaginosis and controls.

Characteristic^∗^	BV women *n* = 144	Control women *n* = 151	*P* value^†^
Age, year	30 (27.33-32)	32 (25-35)	0.177
Familial history of BV, no. (%)	77 (53.5)	37 (24.5)	<0.001
Education, no. (%)			0.408
Primary/secondary school	37 (25.7)	39 (25.8)	
Bachelor's degree	76 (52.8)	70 (46.4)	
Master's/doctoral degree	31 (21.5)	42 (27.8)	
Cigarette smoker, no. (%)			<0.001
Current smokers	26 (18.1)	2 (1.3)	
Never smokers	103 (71.5)	140 (92.7)	
Ex-smokers	15 (10.4)	9 (6)	
Employment status, no. (%)			0.698
Employed	43 (29.9)	42 (27.8)	
Unemployed	101 (70.1)	109 (72.2)	
Monthly family income, no. (%)			0.523
<250 US $	111 (77.1)	121 (80.1)	
≥250	33 (22.9)	30 (19.9)	
Frequency of pregnancy, no. (%)			0.885
0	64 (44.4)	70 (46.4)	
1-2	66 (45.8)	65 (43)	
≥3	14 (9.7)	16 (10.6)	
Menstrual cycle, no. (%)			0.774
Irregular	49 (34)	49 (32.5)	
Regular	95 (66)	102 (67.5)	
Number of sexual partners in the last one month, no. (%)			0.794
0	43 (29.6)	44 (28.9)	
1	95 (66)	103 (68.1)	
≥ 2	6 (4.4)	4 (3)	
Physical activity (MET/h/d)	40.35 (37.03-43)	40.30 (37.67-43.34)	0.599
BMI (kg/m^2^)	26.12 (24.28-28)	24.61 (22.96-26.31)	0.021
Waist circumference (cm)	85 (82-91)	82 (77-89)	0.038
Waist to hip ratio	0.53 (0.50-0.57)	0.51 (0.47-0.55)	0.126

^∗^Values are median (Q1-Q3) unless otherwise noted. ^†^Using Mann–Whitney *U* or *χ*^2^ test, as appropriate. BV: bacterial vaginosis; BMI, body mass index.

**Table 2:** Dietary intake and dietary carbohydrate indices among bacterial vaginosis cases and controls.

Dietary factors^∗^	BV women *n* = 144	Control women *n* = 151	*P* value^†^
Energy intake (kcal/d)	2481 (1985-2982)	2223 (1845-2886)	0.306
Carbohydrate (g/d)	325.7 (245.8-387.6)	307.5 (240.5-373.7)	0.463
Protein (g/d)	76.2 (67.1-90.9)	80.8 (65.9-99.6)	0.234
Fat (g/d)	99.9 (85.1-112.9)	83.5 (69.4-99.5)	0.022
Dietary fiber (g/d)	20.1 (17.1-25.8)	23.8 (18.2-30.8)	0.013
Glycemic index	61.24 (59.3-63.3)	59.49 (57.3-61.8)	<0.001
Glycemic load	190.8 (144.6-234.9)	162.0 (131.4-205.1)	0.069
Insulin index	40.7 (39.2-42.3)	41 (39.4-43.1)	0.675
Insulin load	102381 (76224-121430)	90321 (76072-112340)	0.415

^∗^Values are median (Q1-Q3). ^†^Based on Mann–Whitney test.

**Table 3:** Adjusted odds ratio (OR) estimates and 95% confidence intervals (CIs) for bacterial vaginosis according to the tertile of carbohydrate indices^∗^.

Carbohydrate indices	Tertiles of carbohydrate indices			*P* for trend
	1^st^	2^nd^	3^rd^	
Glycemic index				
No. cases/no. controls	23/50	50/51	71/50	
Base model^†^	1.00 (ref.)	2.10 (1.12-3.95)	2.95 (1.59-5.46)	0.001
Full model ^‡^	1.00 (ref.)	2.10 (1.02-4.31)	2.99 (1.47-6.08)	0.003
Glycemic load				
No. cases/no. controls	42/50	36/51	66/50	
Base model^†^	1.00 (ref.)	0.85 (0.47-1.53)	1.55 (0.90-2.69)	0.102
Full model ^‡^	1.00 (ref.)	1.12 (0.50-2.49)	4.01 (1.22-5.91)	0.029
Insulin index				
No. cases/no. controls	49/50	53/51	42/50	
Base model^†^	1.00 (ref.)	0.95 (0.54-1.68)	0.81 (0.45-1.43)	0.465
Full model ^‡^	1.00 (ref.)	0.78 (0.39-1.55)	0.76 (0.35-1.66)	0.496
Insulin load				
No. cases/no. controls	46/50	43/51	55/50	
Base model^†^	1.00 (ref.)	0.94 (0.53-1.67)	1.17 (0.67-2.04)	0.579
Full model ^‡^	1.00 (ref.)	0.87 (0.39-1.97)	0.83 (0.22-3.10)	0.769
Carbohydrate				
No. cases/no. controls	47/50	43/51	54/50	
Base model^†^	1.00 (ref.)	0.93 (0.53-1.66)	1.16 (0.66-2.03)	0.594
Full model ^‡^	1.00 (ref.)	1.03 (0.45-2.37)	1.46 (0.37-5.86)	0.640
Dietary fiber				
No. cases/no. controls	56/50	68/51	19/50	
Base model^†^	1.00 (ref.)	1.24 (0.73-2.11)	0.36 (0.19-0.69)	0.007
Full model ^‡^	1.00 (ref.)	0.72 (0.36-1.45)	0.22 (0.14-.33)	<0.001

^∗^Logistic regression model. ^†^Adjusted for age. ^‡^Adjusted for age, BMI (Kg/m^2^), WC (cm), cigarette smoker (yes, never, ex-smokers), energy intake (Kcal/d), fat intake (g/d), familial history of BV (yes/no), and physical activity (MET/h/d).

## Data Availability

The data used to support the findings of this study are available from the corresponding author upon reasonable request.
